# Supplementary material for: Identification of photoperiod-induced specific miRNAs in the adrenal glands of Sunite sheep (Ovis aries)
Source: Front Vet Sci. 2022 Jul 22;9:888207. doi: 10.3389/fvets.2022.888207 (PMC9354845; doi:10.3389/fvets.2022.888207)
Supplement: Supplementary file 1 [file Data_Sheet_1.ZIP › Supplementary file/miRDeep2 file.zip!/miRDeep2 file/Know_Structure/SPLP42A1/oar-mir-148a.pdf]

miRBase precursor	: oar-mir-148a
Total read count	: 1956070
oar-miR-148a read count	: 1953281
remaining reads	: 2789

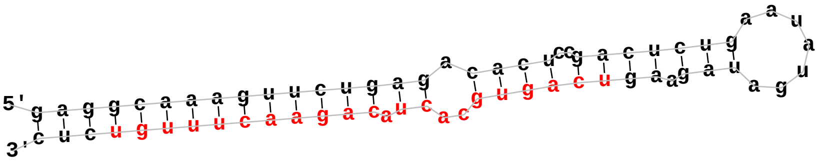

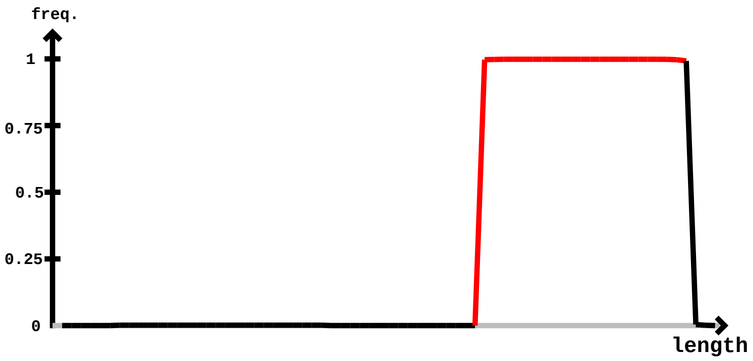


```
                                                oar-miR-148a
5'- gaggcaaaguucugagacacuccgacucugaauaugauagaagucagugcacuacagaacuuugucuc   -3'    exp
    (((((((((((((((.((((..(((((((.......))).))))))))..)).)))))))))))))))   reads      mm         sample
    ....caaaguucugagacacuccga...........................................   7          0          seq
    ....caaaguucugagacacuccgac..........................................   3          0          seq
    ....caaaguucugagacacuccgacu.........................................   74         0          seq
    .....aaaguucugagacacuc..............................................   1          0          seq
    .....aaaguucugagacacuccg............................................   11         0          seq
    .....aaaguucugagacacuccga...........................................   71         0          seq
    .....aaaguucugagacacuccgac..........................................   32         0          seq
    .....aaaguucugagacacuccgacu.........................................   2574       0          seq
    .....aaaguucugagacacuccgacuc........................................   3          0          seq
    .....aaaguucugagacacuccgacucu.......................................   6          0          seq
    .....aaaguucugagacacuccgacucug......................................   3          0          seq
    ......aaguucugagacacuccgacu.........................................   3          0          seq
    ........................................aagucagugcacuacagaacuuugu...   1          0          seq
    ..........................................gucagugcacuacagaacuuug....   3          0          seq
    ..........................................gucagugcacuacagaacuuugu...   82         0          seq
    ..........................................guUagugcacuacagaacuuugu...   1          1          seq
    ...........................................ucagugcacuacagaaA........   1          1          seq
    ...........................................ucagugcacuacagaac........   2          0          seq
    ...........................................ucaguAcacuacagaac........   1          1          seq
    ...........................................ucagugAacuacagaacu.......   1          1          seq
    ...........................................ucaguAcacuacagaacu.......   1          1          seq
    ...........................................ucaCugcacuacagaacu.......   3          1          seq
    ...........................................uUagugcacuacagaacu.......   1          1          seq
    ...........................................ucagugcacuacagaacu.......   190        0          seq
    ...........................................ucagugcacuacagaacuu......   418        0          seq
    ...........................................ucaguAcacuacagaacuu......   1          1          seq
    ...........................................ucagugcacuacagaacuuu.....   2583       0          seq
    ...........................................uUagugcacuacagaacuuu.....   3          1          seq
    ...........................................uUagugcacuacagaacuuug....   13         1          seq
    ...........................................ucagugcacuacagaacuuug....   8736       0          seq
    ...........................................ucagugcacuacagaacuuugu...   1928398    0          seq
    ...........................................uUagugcacuacagaacuuugu...   2733       1          seq
    ...........................................ucagugcacuacagaacuuuguc..   5015       0          seq
    ...........................................uUagugcacuacagaacuuuguc..   8          1          seq
    ...........................................ucagugcacuacagaacuuugucu.   2323       0          seq
```

oar-miR-148a

```
gaggcaaaguucugagacacuccgacucugaauaugauagaagucagugcacuacagaacuuugucuc
...........................................ucagugcacuacagaacuuugucuc    6       0       seq
............................................cagugcacuacagaacuuu.....    2       0       seq
............................................cagugcacuacagaacuuug....    2       0       seq
............................................cagugcacuacagaacuuugu...    1167    0       seq
............................................Uagugcacuacagaacuuugu...    3       1       seq
............................................cagugcacuacagaacuuuguc..    6       0       seq
............................................cagugcacuacagaacuuugucu.    6       0       seq
.............................................agugcacuacagaacuuu.....    1       0       seq
.............................................agugcacuacagaacuuug....    12      0       seq
.............................................agugcacuacagaacuuugu...    1527    0       seq
.............................................agugcacuacagaacuuuguc..    6       0       seq
.............................................agugcacuacagaacuuugucu.    3       0       seq
..............................................gugcacuacagaacuuug....    1       0       seq
..............................................gugcacuacagaacuuugu...    3       0       seq
...............................................ugcacuacagaacuuugu...    3       0       seq
................................................gAacuacagaacuuugu...    1       1       seq
................................................gcacuacagaaAuuugu...    1       1       seq
................................................gcacuacagaacuuugu...    13      0       seq
................................................gcacuacagaacuuuguc..    1       0       seq
```
